# Geomagnetic Shielding Enhances Radiation Resistance by Promoting DNA Repair Process in Human Bronchial Epithelial Cells

**DOI:** 10.3390/ijms21239304

**Published:** 2020-12-06

**Authors:** Xunwen Xue, Yasser F. Ali, Caorui Liu, Zhiqiang Hong, Wanrong Luo, Jing Nie, Bingyan Li, Yang Jiao, Ning-Ang Liu

**Affiliations:** 1 State Key Laboratory of Radiation Medicine and Protection, School of Radiation Medicine and Protection, Collaborative Innovation Center of Radiological Medicine of Jiangsu Higher Education Institutions, Soochow University, Suzhou 215123, China; xuexunwen0218@163.com (X.X.); yasser.ali@azhar.edu.eg (Y.F.A.); caoruiliu@163.com (C.L.); hong1152076509@163.com (Z.H.); luowanrong4596@126.com (W.L.); jingnie@suda.edu.cn (J.N.); bingyanli@suda.edu.cn (B.L.); jiaoyang@suda.edu.cn (Y.J.); 2 Department of Nutrition and Food Hygiene, Soochow University of Public Health, Suzhou 215123, China

**Keywords:** hypomagnetic field, ionizing radiation, DNA damage response, human lung epithelial cells

## Abstract

With the advent of long-duration space explorations, ionizing radiation (IR) may pose a constant threat to astronauts without the protection of Earth’s magnetic field, or hypomagnetic field (HMF). However, the potential biological effects of a HMF on the cellular response to IR have not been well characterized so far. In this study, immortalized human bronchial epithelial cells were exposed to X-rays under either a geomagnetic field (GMF, ~50 uT) or HMF (<50 nT) culture condition. A significant increase of the cell survival rate in HMF after radiation was observed by colony formation analysis. The kinetics of DNA double-strand breaks (DSBs), determined by γH2AX foci formation and disappearance, presented a faster decrease of foci-positive cells and a significantly lower mean number of γH2AX foci per nucleus in HMF-cultured cells than in GMF-cultured cells after radiation. In addition, a γH2AX/53BP1 colocalization assay showed an upregulated DSB recovery rate in HMF cultured cells. These findings provided the first evidence that HMF exposure may enhance the cellular DSB repair efficiency upon radiation, and consequently modulate the genotoxic effects of IR.

## 1. Introduction

From the 1980s to the 2010s, all manned spaceflights have been taken place in low-Earth orbit with the protection of our planet’s geomagnetic field (GMF, about 50 μT). However, the environmental magnetic field in outer space decreases geometrically with the distance from the ground, which exposes the astronauts to space radiation during lengthy voyages outside the Earth’s magnetosphere. This GMF-eliminated environment is defined as a hypomagnetic field (HMF), to represent radiation situations in interplanetary space (6.6 nT) [1], Moon surface (<300 nT) [2], and above the Martian crust (<700 nT) [3]. Although HMF and radiation are the two tightly intertwined ecological factors in outer space, their potential to compromise human health has not yet been fully elucidated.

Radiation-induced DNA lesions increase the cellular risk of malignant transformation that has been identified as one of the most harmful outcomes for human health. Among various cancer types, lung cancer is the largest potential health risk from space travel for both men and women astronauts [4]. At the molecular level, radiation is known to negatively impact DNA integrity. To counteract DNA damage, cells have developed specific mechanisms that locate and repair DNA lesions. These mechanisms consist of a network of cellular proteins involved in DNA damage response pathways, such as cell cycle regulation, DNA repair, and apoptosis [5]. Generally, cells can accommodate moderate DNA damage through different DNA repair processes, but in space flight with the lack of a magnetic field, these DNA repair processes may be interrupted by HMF leading to altered radiation sensitivity.

HMF has also been identified as a significant regulator of physical and behavioral functions on earth. Exposure to the HMF environment may induce a range of biological effects and adaptive alterations, such as interfered embryogenesis and brain function [6,7,8,9], increased occurrences of developmental abnormalities in the newt and Xenopus laevis [10,11], alteration of the circadian rhythm of birds [12] and rats [13], dysfunction in the learning and memory of Drosophila and chicks [14,15], and a reduction in stress-induced analgesia in mice [16,17]. It has been reported that even short-term HMF exposure can reduce the heart rate and increase capillary circulation in humans [18]. At the cellular and molecular levels, in vitro and in vivo studies have shown that biological processes maintaining genomic stability, such as cell cycle progression, tubulin assembly, DNA super condensation, reactive oxygen species (ROS) production, and antioxidant capacity, can be modulated by HMF [19,20,21,22]. It is therefore unsurprising that increasing research attention is being directed toward the biological effects of HMF, to provide knowledge that may be used to protect the astronauts.

Previous studies on radiation exposure and HMF have been usually conducted separately, so data regarding their combined effects remain quite limited. The present study was designed to explore the biological effect of HMF on DNA damage response in human bronchial epithelial cells upon exposure to ionizing radiation. To our knowledge, this is the first experiment that aimed to elucidate the joint biological effect of radiation and HMF on genotoxic endpoints in human lung cells.

## 2. Results

### 2.1. Geomagnetic Shielding Alone Does Not Affect DNA Integrity

**Figure 1:**
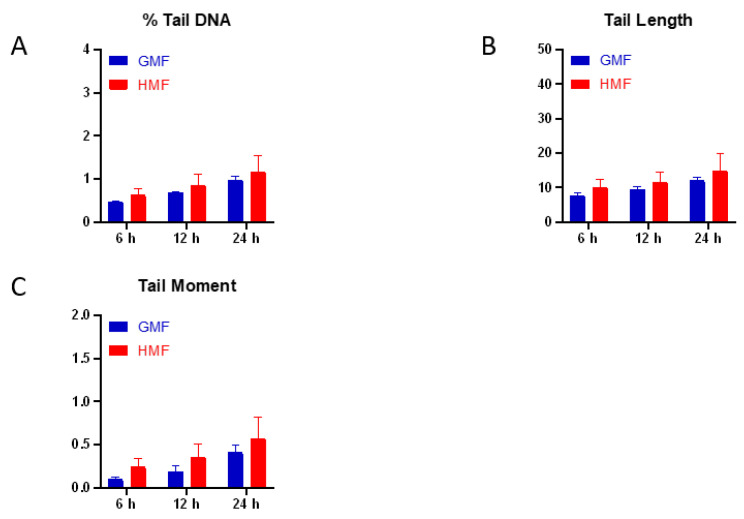
Comet assay results of cells exposed to HMF. The percentage of DNA in tail (**A**), tail length (**B**) and tail moment (**C**) are shown in the panel correspondingly. The results of triplicate samples of three independent experiments are reported together with the SEM for each time-point. Cells in exponential phase of growth were cultured in HMF for 6, 12 and 24 h.

The alkaline comet assay is a sensitive method for measuring DNA lesions (including single and double strand breaks, and base modifications) and detecting repair kinetics in single cells. The amount of DNA migration under electric potential indicates the amount of DNA damage in the cell. We first examined the potential effect of HMF exposure alone on DNA integrity by measuring DNA damage through the alkaline comet assay. We found that exposure to the HMF for 6, 12, and 24 h did not significantly change the average DNA damage indicated by the percentage of DNA in tail, tail length, and tail moment in HMF cultured BEAS-2B cells when compared to GMF control cells (Figure 1). These results suggest that up to 24 h of exposure to HMF has no obvious genotoxic effect in BEAS-2B cells.

### 2.2. Geomagnetic Shielding Enhances Radiation Resistance in BEAS-2B Cells

**Figure 2:**
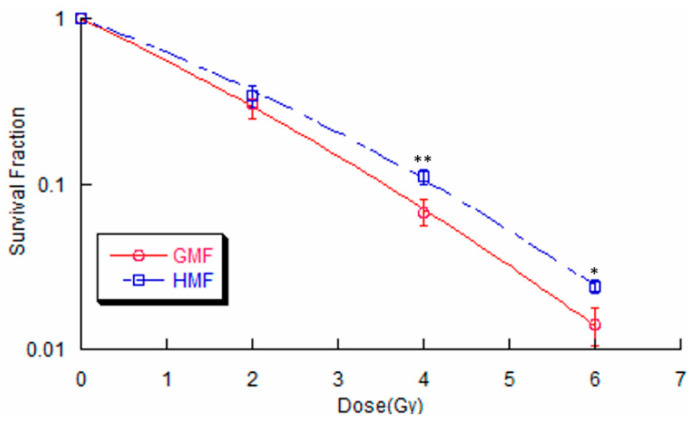
Survival curves of BEAS-2B cells irradiated by a series doses of X-ray under HMF or GMF conditions. Experimental data represent the mean of three paralleled samples from three independent experiments. (* *p* < 0.05, ** *p* < 0.01 compared with the GMF group, ANOVA).

To determine whether geomagnetic shielding alters radiosensitivity in cells, a clonogenic assay was first adopted. As shown in Figure 2, the survival curve was calculated in BEAS-2B cells that were exposed to various doses of X-rays under HMF or GMF culture conditions, respectively. Moreover, it was found that geomagnetic shielding significantly increased the surviving fraction (SF) of BEAS-2B cells after IR exposure. These results indicate that cells cultured in HMF are relatively less susceptible to radiation damage than in regular GMF condition.

### 2.3. Geomagnetic Shielding Accelerates the Decline of IR Induced γH2AX Foci

**Figure 3:**
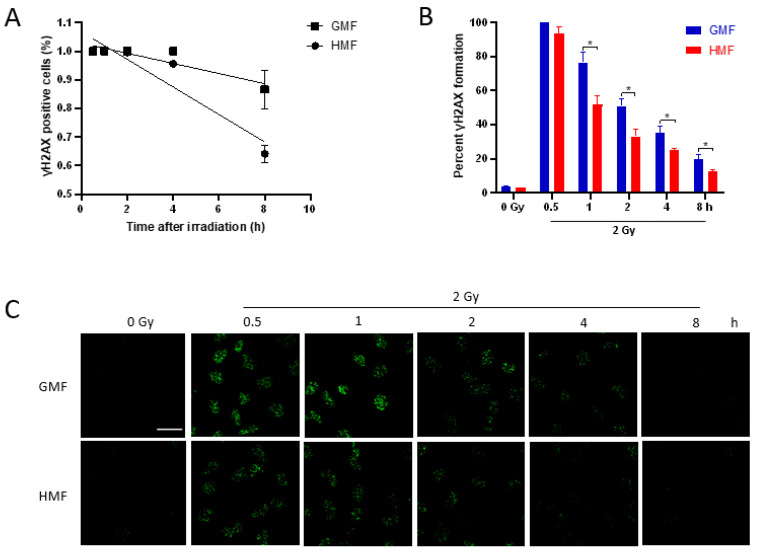
Kinetics of γH2AX foci in BEAS-2B cells irradiated with 2 Gy of X-rays and incubated in HMF or in GMF before and after IR. (**A**) Percentage of cells positive for γH2AX foci, determined by counting 200–250 cells for each experimental point. (**B**) Percentage of γH2AX foci per nucleus; at least 150 cells/time-point were scored for foci. (**C**) Immunofluorescence of γH2AX foci at 0.5, 1, 2, 4, and 8 h after irradiation. Results represent the mean of 3–4 independent experiments ± SEM (* *p* < 0.05, ANOVA), Scale bar = 30 μm.

**Figure 4:**
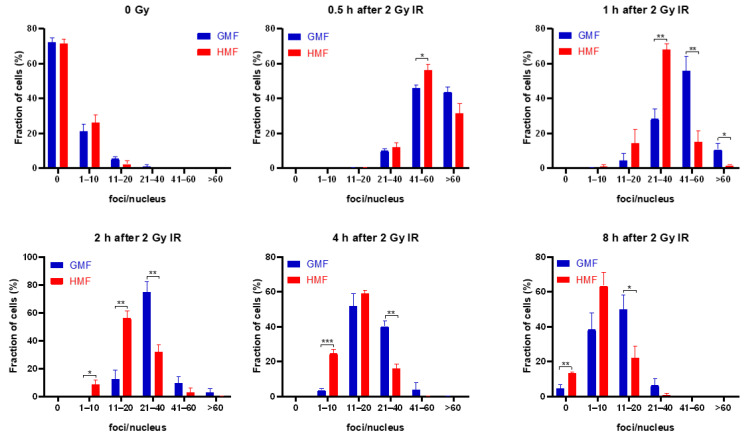
Fraction of cells (% ± SEM) with 0, 1–10, 11–20, 21–40, 41–60, >60 γH2AX foci/nucleus in HMF and GMF cultured cells at indicated time points after exposure to IR. Visualization and image extraction of γH2AX foci was performed by confocal laser microscopy following immunofluorescence staining. After obtaining a distribution of cells with the counted numbers of foci per nucleus, the fraction of cells with appropriate foci number in an indicated range was determined. Results represent the mean of 3–4 independent experiments ± SEM (* *p* < 0.05, ** *p* < 0.01, *** *p* < 0.001, ANOVA).

DNA has been considered as the main cellular target of deleterious effects of IR, exposure of which is followed by many types of DNA damages. DNA double-strand breaks (DSBs) induced by IR are considered the most lethal lesions. Unrepaired or misrepaired DSBs are a serious threat to genomic integrity, and thus associated with cell fate decision, such as apoptosis, necrosis, and senescence. Given the important role of DSB in negatively affecting cell survival, it appears likely that the enhanced radioresistant capacity of BEAS-2B cells due to geomagnetic shielding is the result of downregulated IR-induced DSB level. To test this idea, the presence of nuclear γH2AX foci in HMF or GMF treated BEAS-2B cells was monitored by in situ immunofluorescence before and after irradiation during a repair time of 8 h. Regression analysis was used to fit the data of γH2AX foci-positive cell percentage and best fit was a linear function of the time after irradiation, slopes being α = 0.048 ± 0.005 in HMF (R = 0.95; *p* < 0.01) and α = 0.018 ± 0.005 in GMF (R = 0.92; *p* = 0.001; Figure 3A). By comparing the mean values, the fraction of foci positive cells was significantly lower in HMF than that in GMF at 2 h (*p* < 0.01) and 4 h (*p* < 0.05). In accordance with these data, the increased foci number in HMF at 0.5 h after 2 Gy IR was reached to 93.6% of which increased in GMF control group, comparable with the increasing rate of foci number in GMF (Figure 3B). At later time points, the rate of γH2AX focus loss was significantly different. In fact, the numbers of γH2AX foci in HMF cultured BEAS-2B cells were declined to 51.7% at 1 h, 33.1% at 2 h, 24.9% at 4 h, and 12.4% at 8 h after irradiation, while the cells in GMF group remained 76.5%, 50.9%, 35.3%, and 19.7% of γH2AX foci at 0.5 h after IR, correspondingly (*p* < 0.05 for 1 h, 2 h, 4 h, and 8 h post IR, Figure 3B,C). The mean numbers of foci/nucleus from 1 h to 8 h after IR were also significantly lower in HMF cultured cells compared to GMF cultured control cells (*p* < 0.01 for 1 h and *p* < 0.05 for 2 h, 4 h, and 8 h post IR, Figure S1). The different kinetics of γH2AX foci disappearance in different conditions was more evident by categorising the cells as having 0, 1–10, 11–20, 21–40, 41–60, or more foci/nucleus (Figure 4). In HMF cultured BEAS-2B, cells with 21–40 or more foci/nucleus were significantly reduced starting from 2 h and almost absent at 8 h after irradiation when compared to that of GMF cultured control cells which remained present up to 8 h post IR (Figure 4).

**Figure 5:**
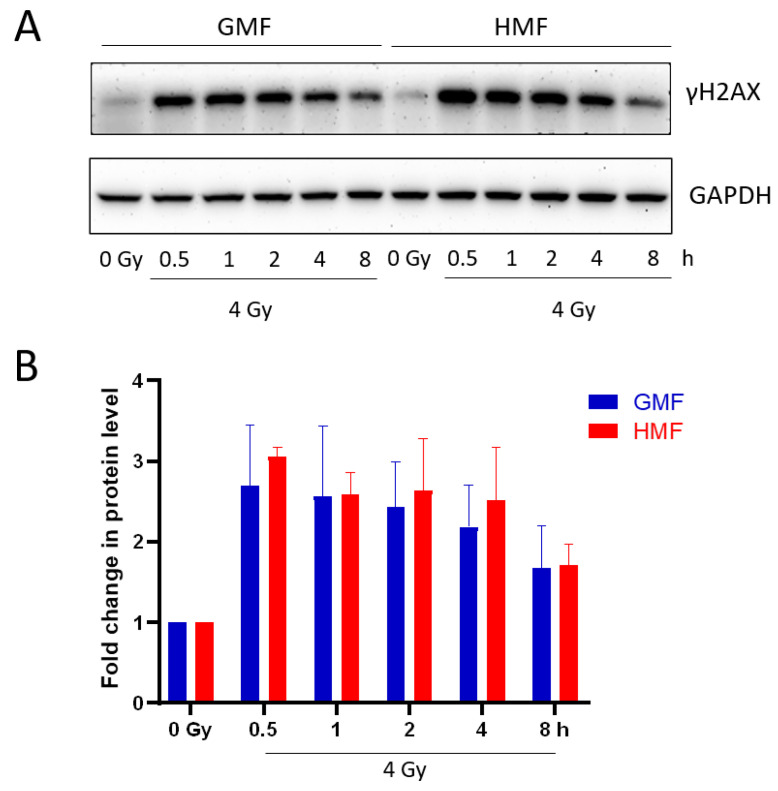
Western blot immunoreactivity of γH2AX. (**A**) Whole cell extracts were prepared at 0, 0.5, 1, 2, 4, and 8 h after 2 Gy exposure. Western blotting was performed with specific antibody. GAPDH as a loading control; (**B**) histograms showing the relative levels of γH2AX in (**A**) experiments. The ratio of γH2AX to Actin at 0 Gy is normalized to “1”. Data represent mean ± SEM (*n* = 3). Data were analyzed using one-way ANOVA.

The cellular level of phosphorylated H2AX was assessed by Western blotting in non-irradiated and irradiated BEAS-2B cells maintained in HMF or GMF. As shown in Figure 5, γH2AX was induced by IR at 30 min after irradiation with a similar expression up to 2 h in both magnetic field conditions. At 4 h after irradiation, γH2AX level slightly decreased in GMF, but persisted in HMF. At 8 h post IR, decreased γH2AX was determined in both HMF and GMF groups. Taken together, the data above suggested that HMF may play an important role in cell recovery from γH2AX foci positive DSB sites after irradiation.

### 2.4. Geomagnetic Shielding Promotes DNA Repair Process at the IR Induced DSB Sites

**Figure 6:**
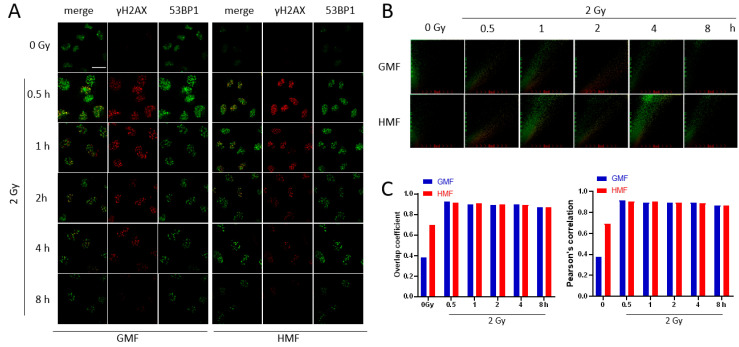
Effects of geomagnetic shielding on IR induced colocalization of γH2AX and 53BP1. (**A**) Representative images from immunofluorescence staining of colocalization between phospho-histone H2AX and anti-53BP1 antibodies in HMF or GMF cultured BEAS-2B cells that were incubated until indicated time post-irradiation and immunofluorescent staining was performed. Scale bar = 30μm. (**B**) 2D correlation histogram of the colocalized γH2AX and 53BP1. (**C**) Colocalization coefficients of γH2AX and 53BP1 immunofluorescent staining in BEAS-2B cells cultured with HMF or GMF were incubated until indicated time post-irradiation. Data were analyzed using one-way ANOVA.

Given the role of HMF in accelerating the decline of γH2AX foci positive DSB sites after IR, it was pertinent to study the effects of HMF on DSB repair processing. In the present work, immunofluorescence staining was utilized to determine the colocalization of γH2AX with p53 binding protein 1 (53BP1), which was widely accepted as a marker for DSB repair [23] (Figure 6A). The colocalization factors between γH2AX and 53BP1 foci were assessed by both Pearson correlation and Mander’s overlap coefficients. As shown in Figure 6B,C, two of the colocalization factors were both upregulated following IR exposure, indicating an activated DSB repair process. In support of HMF mediated radioresistance enhancement, considering the accelerated decline of γH2AX foci, the similar proportion of double labeled foci after IR in HMF and GMF groups (Figure 6B,C) suggested that the DSB repair activity was more efficiently stimulated in HMF cultured cells compare to GMF control cells after IR exposure.

## 3. Discussion

The interplanetary magnetic field that the crew may be exposed to during manned flight is 10^3^–10^4^ times less than the regular GMF on the Earth surface, i.e., HMF. The importance of HMF in outer space lies in its impact on infiltration of intensive fluxes of radiation into the spacecraft, and most of which is blocked by GMF during the low-Earth orbit flights. Since radiation is known to induce genetic lesions to the cells and so increase human cancer risk, it is essential to understand the biological effect of HMF in combination with IR in terms of DNA damage and repair. In the present work, we revealed that, despite the fact that HMF alone showed no obvious effect on DNA integrity, it clearly enhanced the radiation resistant capacity of human bronchial epithelial cells by accelerating the decline of γH2AX foci after radiation. The result implied that the geomagnetic field shielding may stimulate cells to recover from IR induced DSB lesions. To confirm the inference, we further demonstrated that HMF could promote cellular DSB repair activity in response to IR by keeping the similar proportion of colocalization of γH2AX and 53BP1, compared with the GMF control.

The effects of HMF on biological systems vary according to magnetic shielding approach, remanence intensity being used, and duration of the exposure [8]. The phosphorylation of histone H2AX on Ser139, which induces the designated γH2AX, has been used as an indicator of DSB. A recent study reported that γH2AX foci were markedly upregulated by magnetic deprivation in preputial skin fibroblasts [24]. In the present study, HMF exposure brought about marginal alteration in spontaneous DNA damage (Figure 1) and γH2AX foci formation (Figure 3). The results are in accordance with a previous report on the repression of endogenous DNA oxidative damage by reactive oxygen species (ROS) in HMF cultured human neuroblastoma cells [25], suggesting a minor genotoxic effect of HMF on DNA integrity.

Using the well-established colony formation assay, an increased cell survival rate in HMF group following IR treatment was observed (Figure 2), indicating an enhanced radiation resistant capacity under HMF condition. Since DSB lesions are considered the most lethal type of DNA damage induced by radiation, this increased cell survival rate in HMF cultured cells might be resulted from either a decreased DSB induction or/and enhanced DSB repair capacity. To distinguish between these possibilities, a γH2AX foci kinetics assay was performed to monitor the functioning of the DSB response machinery [26]. Our data showed that geomagnetic shielding resulted in a significant decline in γH2AX foci number per nucleus from 1h to 8h post IR, compared to the GMF controls. While at 30 min after IR, the maximum yield of γH2AX foci between HMF and GMF culture groups showed no statistical difference (Figure 3B). These results suggested a strengthened DSB recovery efficiency due to the deprivation of geomagnetic field, as indicated by the increased cell survival rate. 

Unlike γH2AX foci kinetics assay, the result from whole cell extracts detected by western blotting (Figure 5) did not show significant changes between experimental and control groups. This may be accounted by the fact that multiple mechanisms have been proposed for eliminating γH2AX when DNA repair completes, including removal by histone exchange or dephosphorylation by a protein phosphatase [27,28]. Thus, the histone H2AX may remain phosphorylated after foci disassembly and the kinetics of disappearance of γH2AX foci may be different from its cellular amount. Moreover, the level of γH2AX signal detected by Western blotting could be explained by the saturation of phosphorylated H2AX targeted for degradation [29,30] and to the contribution of apoptotic DNA fragmentation.

DSBs can be repaired either through the error-prone Non-homologous end-joining (NHEJ) pathway, or through homologous recombination (HR) in the presence of a DNA donor template with high fidelity [31]. The modification of H2A and H2AX triggers the binding of early phase DSB repair proteins, amongst which 53BP1 plays an important role in the DSB repair initiation and NHEJ/HR pathway selection [32]. The presence of 53BP1 at the DSB normally results in the recruitment of proteins involved in NHEJ repair pathway, and inhibition of BRCA1 takes part in the HR repair pathway [33,34]. The concurrence of γH2AX and 53BP1 at the DSBs initiates an on-site DSB repair process, which can be detected by colocalized antibody binding [23,35]. The colocalization of 53BP1 and γH2AX foci in cells upon radiation has been found similar to the DSBs repair sites [24,36]. In particular, the phosphorylation of H2AX resulting from DNA damage may enhance the interaction between γH2AX and 53BP1, leading to an increased accumulation of 53BP1 foci at the sites of DSB in IR exposed cells. Notably, our results presented a similar colocalization factor in IR exposed cells under both HMF and GMF culture conditions (Figure 6B,C), indicating a higher ratio of 53BP1/γH2AX co-stained site among total γH2AX positive foci in HMF cultured cells, when taking account of the lower level of γH2AX foci in HMF cultured cells at 1–8 h after IR. Taken together, these data suggested a proficient repair of the IR-induced DSB damage in HMF cultured cells. However, the detailed mechanisms underlying its tumorigenesis are yet to be further elucidated. 

In summary, our study has established, for the first time, that HMF can take part and interact with IR-induced DNA damage and DNA repair process, so as to provide an enhanced radiation resistant capability in human lung cells. As a previously unidentified regulator of radiation sensitivity, the new biological function of HMF may be potential in future astronaut’s radiation protection as well as in human cancer research.

## 4. Materials and Methods

### 4.1. Cell Culture

Human bronchial epithelial cell line BEAS-2B purchased from American Type Culture Collection were maintained in Dulbecco’s modified Eagle’s medium (DMEM; Gibco, Grand Island, NY, USA) supplemented with 10% (*v*/*v*) fetal bovine serum (FBS; Gibco, Grand Island, NY, USA), 100 unit/mL penicillin and 100 mg/mL streptomycin and were grown in a monolayer in petri dishes (Corning Inc., Corning, NY, USA) and the medium was replaced every two days. The cells were used within 10–20 passages according to the requirements of ATCC, and were routinely tested for mycoplasma using mycoplasma detection kit (Beyotime Biotech, Haimen, China).

### 4.2. The HMF Conditions

HMF was achieved by a permalloy magnetic shield box (the 710th Research Institute of China Shipbuilding Industry Corporation, Yichang, China), and the residue magnetic field inside the magnetic shielding box was relatively uniform. The HMF-exposed cells were cultured within the shielding box where the residue magnetic field was lower than 50 nT. The shield box was put in a cell incubator (Thermo Fisher Scientific, Waltham, MA, USA) equipped with a circulating fan (Thermo Fisher Scientific, Waltham, MA, USA) to ensure the optimal conditions of cell culture (5% CO_2_, 37 °C). Cells of GMF control were cultured in a regular cell incubator (Thermo Fisher Scientific, Waltham, MA, USA), where the magnetic field was about 45 μT (i.e., GMF). The intensity of magnetic field was measured by a gaussmeter (Model BLD-630, Boland Magnetoelectric Technology, Beijing, China).

### 4.3. Irradiation

Cells were plated in Petri dishes and cultured for 24 h before irradiation and subsequently irradiated with X-rays, which were generated using an X-rays instrument (RS 2000 Xray Biological Irradiator, Rad Source Technologies, Suwanee, GA, USA) equipped with a tungsten target (160 kVp, 25 mA). The dose rate was 1 Gy/min. All irradiation treatments were carried out at room temperature.

### 4.4. Alkaline Comet Assay

The alkaline comet assay was performed to detect DNA damage at the single-cell level. Briefly, cells were collected by trypsinization and centrifugation at indicated time points after HMF condition culture and mixed with low melting point agarose to prepare a cell suspension in 0.1% agarose/phosphate-buffered saline (PBS). After gelation of the agarose, the cells were lysed. The resultant DNA samples were electrophoresed at 1 V/cm for 30 min in 0.3 M NaOH and 1 mM ethylenediamine-N,N,N’,N’- tetraacetic acid solution. After the DNA was stained with SYBR Green I, immunofluorescence images were captured on a fluorescence microscope. DNA damage was analyzed using the Comet software (Comet Assay IV, Perceptive Instruments, Suffolk, UK). At least 100 comets from each gel were analyzed. Tail length indicates the pixel length of the comet tail. The tail percentage indicates the percentage of tail content relative to comet content. The tail moment was calculated as follows:

Tail moment = (the distance between the center of the comet head and the center of the comet tail) × (Tail percentage)/100

### 4.5. Cell Survival Assay

The SF was measured using the colony formation assay to assess radiation sensitivity. Cells were inoculated into triplicate 60 mm plastic dishes to produce 20–100 colonies per dish post X-ray (0, 2, 4, 6 Gy) radiation. After 14 days of incubation, colonies were fixed with 20% methanol and stained with 0.2% crystal violet. Triplicate dishes of each dose point colony consisting of more than 50 cells were counted under a stereomicroscope. The SF at each dose point was determined as the ratio of live colonies in the treated dish relative to the number in the untreated/control.

### 4.6. Immunofluorescent Staining

Immunofluorescence staining was carried out as previously described [37]. Primary mouse anti-γH2AX antibody (1:400, sc-517348, Santa Cruz Biotech, Dallas, TX, USA) and rabbit-anti-53BP1 antibody (1:300, ab21083, Abcam, Cambridge, MA, USA) were used. Alexa Fluor 555 –labelled donkey anti-mouse secondary antibody (1:1000, A0460, Beyotime Biotech, Haimen, China) and Alex Fluor 488 –labelled goat anti-rabbit secondary antibody (1:1000, A0423, Beyotime Biotech, Haimen, China) were used for visualization. Cell nuclei were stained with 4’, 6-diamidino-2-phenylindole (DAPI, Sigma, Milwaukee, WI, USA). Imaging of γH2AX foci was performed using a confocal microscope (Olympus FV1200, Tokyo, Japan) with a ×40 oil objective. At least 150 cells were scored randomly from 5 to 10 fields. The number of γH2AX foci per cell and the percentage of γH2AX foci–positive cells (at least 5 foci/nucleus) were used as the indicators of DNA damage. Each experiment was independently repeated three times.

The results from colocalization experiments for 53BP1 and γH2AX foci are quantitatively presented in terms of the colocalization factor (Pearson’s correlation coefficients and Manders’ overlap coefficients) [38], which was analyzed using the Image Pro Plus 6.0 software (Media Cybernetics, Silver Spring, MD, USA).

### 4.7. Western Blotting

Cells were harvested and lysed using RIPA buffer. Samples were sonicated and centrifuged at 12,000× *g* for 15 min at 4 °C. The concentration of total protein was determined by using DC Protein Assay Kit I (Bio-Rad, Richmond, CA, USA). Then, the samples were denatured at 100 °C for 5 min. Total proteins were separated by 12% SDS-PAGE and transferred to a hybond nitrocellulose membrane (Amersham Biosciences, Pascataway, NJ, USA). The membrane was blocked with 5% nonfat milk powder (A600669, Sangon Biotech, Shanghai, China) in Tris-buffered saline which consisted of 20 mM Tris-HCl (1115GR500, Biofroxx, Einhausen, German) and 150 mM NaCl (10019318, Sinopharm, Shanghai, China) adjusted pH to 7.5 by using HCl. The membrane was then hybridized overnight with primary antibodies γH2AX (1:1000, 9718S, Cell Signaling Technology, Beverly, MA, USA), GAPDH (1:1000, RLM3029, Ruiyingbio, Suzhou, China), which were then detected with horseradish peroxidase-conjugated anti-IgG for 2 h at room temperature and visualized with an ECL kit (Millipore, Billerica, MA, USA). Protein expression levels were normalized to the loading controls basing on their intensity analyzed with Image J (Version 1.50i, National Institutes of Health, Bethesda, MD, USA). The target γH2AX/GAPDH value obtained from 0 Gy control cells was designated as “1”.

### 4.8. Statistical Analysis

All computations were performed with GraphPad Prism software (Version 8.0, GraphPad, La Jolla, CA, USA) for Windows. Data are presented as the means ± standard errors of the mean (SEM). After the normality test, statistical analysis was performed with analysis of variance (ANOVA) between HMF exposure and GMF exposure groups. *p* < 0.05 was considered statistically significant.

## Supplementary Materials

Supplementary materials can be found at https://www.mdpi.com/1422-0067/21/23/9304/s1. Figure S1: Mean number of γH2AX foci per nucleus in BEAS-2B cells irradiated with 2 Gy of X-rays and incubated in HMF or in GMF before and after IR.

## Abbreviations

DSB	DNA double-strand break
GMF	Geomagnetic field
HMF	Hypomagnetic field
HR	Homologous recombination
IR	Ionizing radiation
NHEJ	Non-homologous end-joining
53BP1	p53 binding protein 1
ROS	Reactive oxygen species
SF	Surviving fraction
