# Supplementary material for: Geomagnetic Shielding Enhances Radiation Resistance by Promoting DNA Repair Process in Human Bronchial Epithelial Cells
Source: Int J Mol Sci. 2020 Dec 6;21(23):9304. doi: 10.3390/ijms21239304 (PMC7730591; doi:10.3390/ijms21239304)
Supplement: Supplementary file 1 [file ijms-21-09304-s001.pdf]

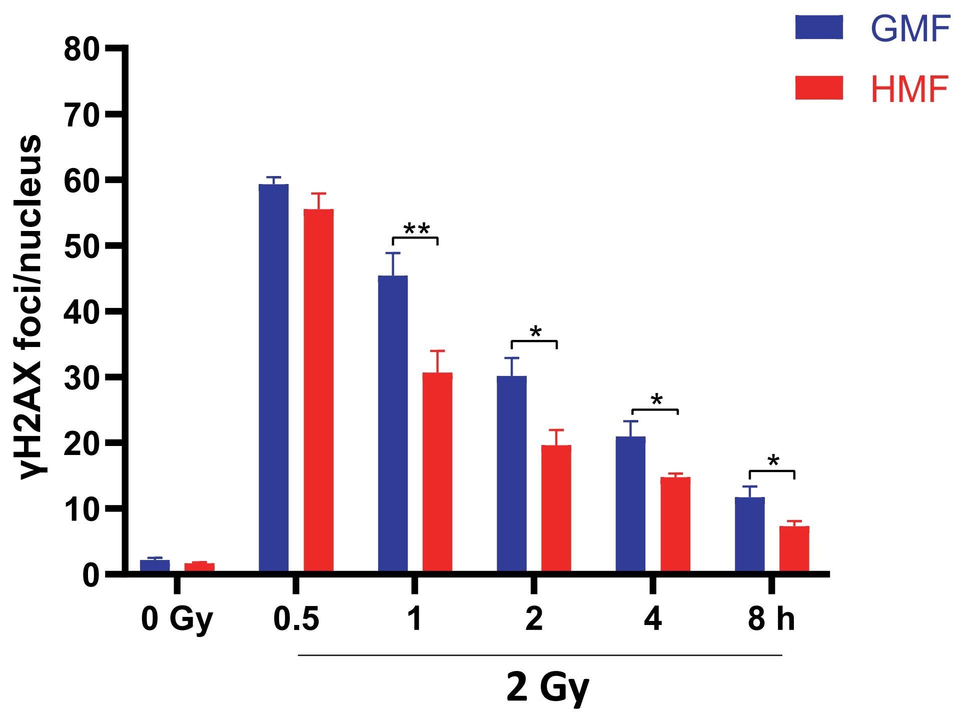

Figure S1. Mean number of γH2AX foci per nucleus in BEAS-2B cells irradiated with 2 Gy of X-rays and incubated in HMF or in GMF before and after IR.
